# Comparison of the Effect of Cumin Cyminum and Nettle Oral Drops on the Breast Milk Sufficiency Indicators in New Mothers

**DOI:** 10.18295/squmj.3.2024.027

**Published:** 2024-05-27

**Authors:** Fatemeh Farshad, Elahe Sadeghi Sahebzad, Masoomeh Kheirkhah, Mahnaz Shafi Khani, Elham Azmoude

**Affiliations:** 1 School of Dentistry, Tehran University of Medical Sciences, Tehran, Iran; 2 Department of Midwifery and Reproductive Health, School of Nursing and Midwifery, Iran University of Medical Sciences, Tehran, Iran; 3 Midwifery & Reproductive Health, Iran University of Medical Sciences, Tehran, Iran; 4 Healthy Ageing Research Centre, Neyshabur University of Medical Sciences, Neyshabur, Iran

**Keywords:** Cumin, Nettle, Breastfeeding, Iran

## Abstract

**Objectives:**

This study aimed to compare the effects of green cumin (Cuminum cyminum) and nettle (Urtica dioica L.) oral drops on the indicators of breast milk adequacy in lactating mothers. Due to the presence of phytoestrogens in the combinations of cumin and nettle, it is stipulated that they may have milk-increasing properties.

**Methods:**

A triple-blind, randomised, controlled clinical trial was conducted on 117 lactating mothers who had given birth to healthy infants aged 10–15 days and who received cumin oral drops (n = 39), nettle oral drops (n = 39) or placebo (n = 39) from August 2020 to March 2021. The participants were recruited from a regional public health care centre affiliated with Iran University of Medical Sciences of Tehran, Tehran, Iran. The 3 study groups received 15 drops thrice a day for 4 weeks. Infant weight, breastfeeding frequency, number of wet diapers, diaper weight and frequency of infant defecation were evaluated before and after the intervention.

**Results:**

At the beginning of the trial, no statistically significant differences were observed between the 3 groups for infant weight (*P* = 0.891), breastfeeding frequency (*P* = 0.921), number of wet diapers (*P* = 0.783), diaper weight (*P* = 0.841) and frequency of infant defecation (*P* = 0.898). However, following the intervention, the mean scores of all indicators were significantly higher in the experimental groups than in the placebo group (*P* <0.001). In addition, all the indicators in the cumin group increased significantly compared to those in the nettle group (*P* <0.001).

**Conclusion:**

Considering the effectiveness of cumin and nettle drops in increasing milk and the availability of these native plants in Iran, it is suggested that they, especially cumin, be used postpartum to increase breast milk production.

**Advances in Knowledge**

- *This study highlights that green cumin is effective in improving the indicators of breast milk adequacy in lactating women and that green cumin is more effective than nettle oral drops in this respect.*

**Application to Patient Care**

- *This study shows that the consumption of cumin or nettle oral drops increases infant weight, breastfeeding frequency, number of wet diapers, infant defecation frequency and diaper weight compared to the placebo group. These indicators were also significantly higher in the cumin group than in the nettle group.*

Breastfeeding plays a major role in infants attaining optimal growth during the first year of their lives.^1^ Breastfeeding is also one of the best ways to ensure child health and survival.^1^ Its effects on maternal health are also well documented.^2^^,^^3^ In addition to beneficial effects on the physical health of mothers and infants, exclusive breastfeeding promotes frequent interaction between mothers and infants, which is vital for the brain development of babies.^4^

Various studies have shown that the percentages of exclusive breastfeeding in the first 6 months differs across cultures.^5^^,^^6^ The prevalence of exclusive breastfeeding is 25% in Africa, 31% in Latin America, 45% in Asia, 13.9% in the USA and 13.8% in Canada.^5^ A study conducted in Iran reported the prevalence of exclusive breastfeeding at 90% in the first year and 57% in the second year after birth. The rates of exclusive breastfeeding in the rural and urban areas of Iran were 5% and 31%, respectively. This study found that mothers’ perception of breast milk insufficiency is one of the most common reasons exclusive breastfeeding is discontinued in the first 6 months in Iran.^6^

Many lactating women seek drugs or non-drug therapy to increase breast milk production.^7^ Drugs that enhance breast milk production are metoclopramide, oxytocin, domperidone and chlorpromazine. The side effects of these drugs include tremors, slow movement, acute dystonic reactions and weight gain.^8^^,^^9^ These medicines are not widely used due to these side effects.^10^

Therefore, women are seeking alternative non-chemical treatments to increase milk production. There are some herbal medicines or substances that initiate, maintain and enhance breast milk production, such as asparagus, dill, parsley, black cumin, alfalfa, galega fenugreek, fennel (Foeniculum vuglare) and nettle.^11^–^17^

Green cumin (Cuminum cyminum) has effective terpenes called karven, myrcene, limonene and alpha- and beta-pinene.^18^ It probably enhances breast milk production by increasing prolactin in the serum of lactating women. In addition to increasing breast milk production, green cumin acts as a pain killer and an anti-spasmodic agent. This herb is used to treat flatulence in today’s medicine.^19^

Another common herbal medicine that increases breast milk production is nettle (Urtica dioica L.). The active ingredients of this plant include polysaccharides, phytosterols, flavonoids and triterpenic acids, which increase prolactin and thus enhance milk production. This herb contains tannin, mucilage and formic acid, phytosterol, potassium and calcium nitrates, iron compounds and a glucoside that causes redness of the skin.^18^ The effect of this herb in the enhancement of breast milk production may be related to an increase in oestrogen levels. Oestrogen apparently increases prolactin receptors, raises prolactin levels and leads to increased milk production by acting directly on the mammary glands.^20^

Few studies have investigated the effects of these 2 medicinal plants in increasing breast milk production.^9^^,^^21^–^23^ In this regard, the results of a randomised clinical trial conducted by Özalkaya *et al*. in Iran showed that consumption of commercially available herbal mixture tea containing nettle and 5 other herbs significantly increased breast milk production in mothers with preterm infants.^21^

To date, no study has compared the breastfeeding outcomes of these two herbal medicines. Therefore, this study aimed to compare the effects of green cumin and nettle on the indicators of breast milk adequacy in lactating mothers.

## Methods

This triple-blind clinical trial was conducted from August 2020 to March 2021 and participants were recruited from a regional public healthcare centre affiliated with Iran University of Medical Sciences of Tehran, Tehran, Iran. This centre is one of the largest centres in the south of Tehran with a large population that provides a range of maternal and child health services. The participants were selected through convenience sampling and comprised lactating women with healthy 10- to 15-day-old infants. Participants were assigned into two experimental groups and one control group.

A pilot study was done to determine the sample size. With the assumption that α = 0.05, β = 0.2, a confidence level of 95%, a test power of 80% and an attrition rate of 10%, the sample size was determined to be 39 in each group according to the below formula:


$$\begin{array}{c} \text{n}=\frac{2\sigma^{2}\;({\text{z}}_{1}-\frac{\alpha }{2}+{\text{z}}_{1-\beta })}{{\left(\mu_{1}-\mu_{2}\right)}^{2}}\\ \sigma^{2}={\text{S}}_{1}^{2}+{\text{S}}_{2}^{2} \end{array}$$

The participants were considered eligible for the study if they were multigravida, had healthy 10- to 15-day-old term-born infants (37–42 weeks gestation), had been exclusively breastfeeding and consented to the study procedures. Women who used other drugs to increase milk supply or did not exclusively breastfeed their infants were excluded. In addition, women with underlying diseases, untreated active tuberculosis, HIV/AIDS, asthma, coagulation disorders, cardiovascular disease and diabetes, hypertension and gastrointestinal diseases and drug or alcohol addiction; who took special medications such as phenobarbital and ergotamine; and who had breast problems such as abscess, nipple indentation and breast cancer were excluded.

Two questionnaires were used for data collection: (1) a maternal-infant information questionnaire (this scale included demographic questions about age, mother’s weight, education levels of mother and father, economic status, parity and type of delivery); and (2) a breast milk sufficiency indicators form (this form was taken from Ghasemi *et al*.’s study.^24^ It evaluates the adequacy of breast milk by measuring the indicators of the feeding frequency—number of wet diapers, frequency of defecation and diaper weight. In the present study, this form was filled daily by mothers).

In addition, growth parameters including weight, height and head circumference of the infants were measured with a weighting scale (Seca, Hamburg, Germany), tall-meter table and tape measure by the researcher, respectively. Participants were randomly assigned to two experimental groups (receiving cumin or nettle oral drops) and a control group (placebo drop) using Microsoft Excel (Microsoft, Redmond, Washington, USA).

All 3 types of drops were manufactured by a pharmaceutical company that provided the oral drops and were labelled only with the randomisation codes (A, B and C). All drops were in the same form in sterile containers. The placebo drop was tasteless, odourless, green and had no milk-enhancing properties. It was also identical to the other herbal drops in appearance and contained sterile water. Cumin drops had the registration number 1624 and nettle edible drops the registration number 1228018596 of the Iran Food and Drug Organization. All participants were administered 15 drops three times per day for 4 weeks. The researcher, evaluator and participants in the 3 study groups were unaware of the type of drops used.

The study variables were measured 2 days before the intervention. At first, the weight of all infants was measured with a scale (Seca) by a trained researcher after changing the diaper, before breastfeeding and without any clothes on the infants. Then, the number and weight of wet diapers per day, the frequency of defecation and feeding frequency were recorded by the participants at home for 2 days using the breast milk sufficiency indicators form. The participants were encouraged to fill out the study form accurately.

According to the assigned group, each participant was provided with oral drops, 3 packs of diapers and a study form to record the indicators of breast milk sufficiency for the first 2 weeks. According to the checklist form, the mothers had to change the diaper every 6 hours and record the number of feedings, the frequency of defecation and the number and weight of wet diapers daily. Wet diapers were collected daily and weighed using a scale at the end of each day.

Follow-up phone calls were made every 3 days to ensure the accuracy of data collection and check for any possible complications. The researcher strongly recommended that the participants complete the forms accurately. In addition, the contact number of the researcher was given to all the participants to contact if needed.

After the first 2 weeks, the participants were referred to the health centre, and the study form was taken and assessed by a trained researcher. Then the participants were given oral drops, 3 packs of diapers and a study form for another 2 weeks.

After the end of the fourth week, the participants and their infants returned to the health centre. The study forms were again received, and the weight of the infants was measured on the previous scale by the researcher after changing the diaper.

The statistical package for the social sciences (SPSS), Version 22 (IBM Corp., Armonk, New York, USA) was used to analyse the data, the Kolmogorov-Smirnov test was used to determine the variable distributions and the Chi-squared test and the one-way analysis of variance (ANOVA) were used to evaluate the demographic and clinical characteristics among the study groups. Additionally, the one-way ANOVA was used to compare changes in the parameters at baseline and the end of the treatment between the study groups. *P* values less than 0.05 were considered statistically significant.

The study protocol was approved by the ethics committee of Iran University of Medical Sciences (IR. IUMS.REC 1396.32068). Written informed consent was obtained from all couple participants before and after the study enrolment in accordance with the Declaration of Helsinki. Additionally, the participants were free to withdraw from the study at any time without providing a reason. All participants were given the researcher’s phone number to report any side effects for mothers and babies. This study was registered in the Iranian Registry of Clinical Trials (IRCT20180609040022N1).

## Results

**Figure 1:**
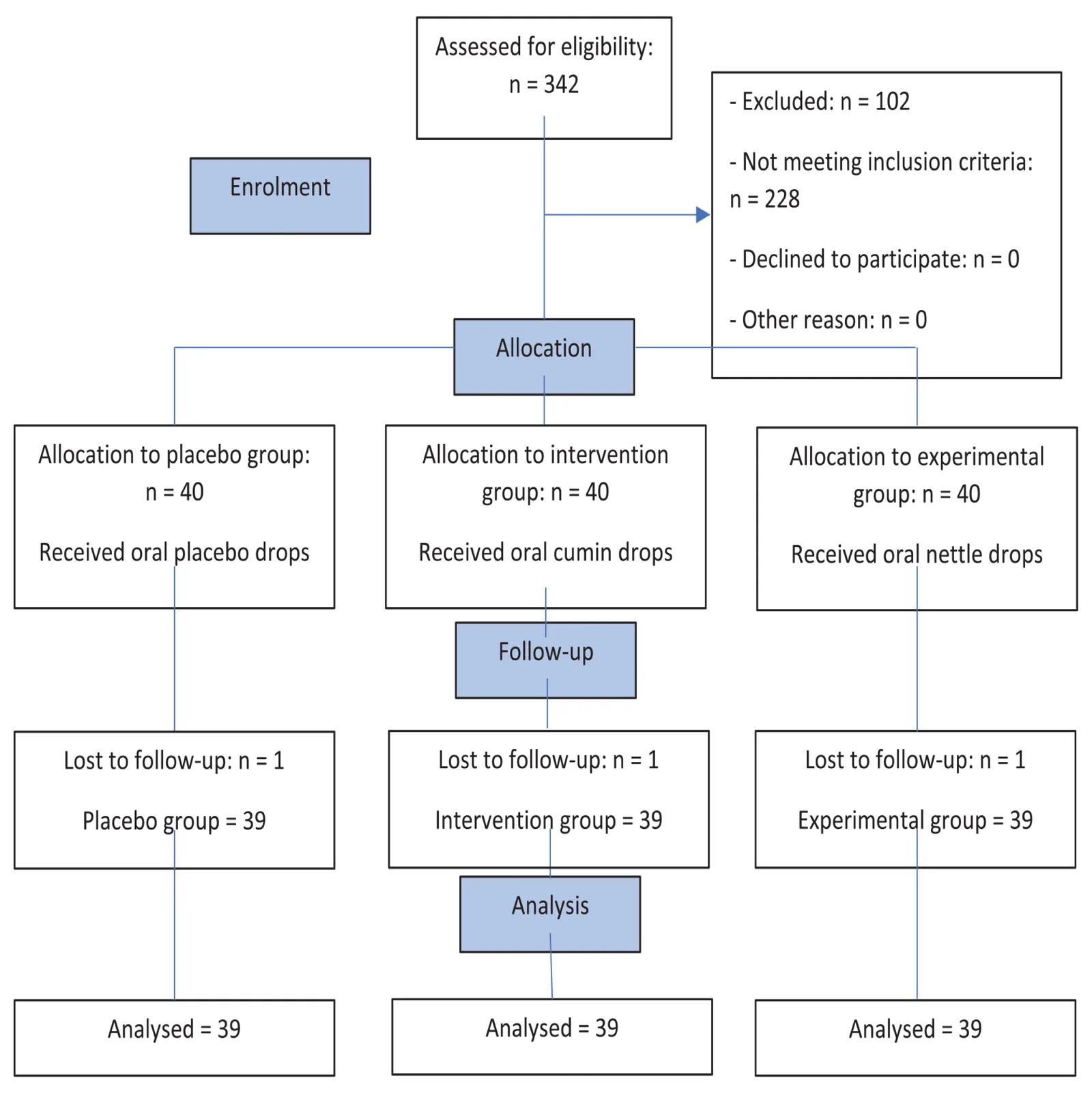
Flowchart showing the selection of participants and group allocations.

The study was conducted on 117 lactating women in 3 groups of intervention and placebo with 39 people in each group [Figure 1]. Throughout the research, the flow diagram depicted the inclusion and exclusion criteria for the participants according to the CONSORT statement. During the study, 3 out of 117 participants were excluded from the study (1 woman from each group).

The average maternal age was 25.8 ± 7.06, 27.3 ± 4.48 and 29.05 ± 6.73 in the cumin, nettle and placebo groups (*P* = 0.293), respectively. The majority of the participants in the 3 groups had a high school education (*P* = 0.343). No significant differences were observed between the 3 groups in terms of other demographic and clinical characteristics (*P* >0.05) [Table 1].

Before the intervention, no significant differences were observed between the groups in terms of infant weight (*P* = 0.891) [Table 2]. However, at the end of the intervention, there were significant differences between the 3 groups in terms of infant weight (*P* <0.001). Infant weight was significantly higher in the green cumin group compared to the nettle and the placebo groups (*P* <0.001). Infant weight was also significantly higher in the nettle group than in the placebo group (*P* <0.001) [Table 3].

Furthermore, there were no significant differences in breastfeeding frequency, number of wet diapers, infant defecation frequency and diaper weight before the intervention between the 3 study groups (*P* = 0.921, *P* = 0.783, *P* = 0.898 and *P* = 0.841, respectively) [Table 2]. After the intervention, significant differences were found between the 3 groups in all the parameters (*P* <0.001). The highest score was observed in the green cumin group [Table 3].

The post-hoc analysis showed significant differences between the green cumin and the nettle groups (*P* = 0.001, *P* = 0.001, *P* = 0.041 and *P* = 0.021). In addition, the post-hoc analysis in both experimental groups indicated significant differences between them and the control group in all the variables (*P* <0.001). No side effects were reported during the intervention.

## Discussion

To the best of the authors’ knowledge, this is the first study to compare the effects of cumin and nettle oral drops on the indicators of breast milk adequacy. In the current study, consumption of cumin or nettle oral drops increased infant weight, breastfeeding frequency, number of wet diapers, infant defecation frequency and diaper weight, compared to the placebo group. These indicators were also significantly higher in the cumin group compared to the nettle group after intervention.

Based on evidence, green cumin has oestrogenic properties. The active ingredients of this herb include a type of terpene called carvone or myrcene, limonene and alpha- and beta-pinene, which increases milk production. Green cumin increases milk production possibly by increasing the oestrogen and prolactin levels.^25^^,^^26^

However, based on many studies, herbal galactogogues increase milk production despite no increase in prolactin levels.^27^–^29^ This indicates the presence of alternative mechanisms influenced by galactogogues. In Liu *et al*.’s study, herbal galactogogues regulated the expression and function of the aquaporins receptors of mammary glands and increased milk secretion in rats.^29^ Consequently, there may be other mechanisms for the effectiveness of nettle and cumin in increasing milk production.

However, the results of the current study are consistent with those of Humphries and Reynolds who found that nettle increased the production of milk, oestrogen and prolactin.^22^ Zuppa *et al*.’s study investigating the effect of milk supplements (fennel, cumin and dill) on milk production found that the group receiving these galactogogues experienced an increase in milk volume, frequency and weight of infants compared to the placebo group.^9^ Similarly, Özalkaya *et al*. evaluated the effect of a herbal tea mixture containing stinging nettle on milk production and serum prolactin levels in lactating women with preterm infants.^21^ The increase in milk production was higher in the herbal tea mixture group from the 1^st^ to the 7^th^ day. However, infant weight in the group receiving this galactogogues mixture was not significantly higher than those in the galactogogues group compared with after 7 days. It is important to note that this composition does not affect the serum prolactin levels of mothers.^21^ The differences between these results and the present research can be due to the shorter duration of the intervention and the use of a combination of plants in the Özalkaya *et al*.’s study.^21^ In an animal study, Maleknezhad *et al*. investigated the effect of the medicinal plant nettle on the growth indicators of young beluga fish. The growth indicators of the fish fed with the diet containing nettle increased significantly compared to fish in the control treatment group.^23^

Based on the results of the current study and given their availability, low prices and lower side effects, these two herbal medicines are recommended for use as an aid to increase breast milk production. However, due to the limited number of trials in this area, further research with larger sample sizes and longer intervention durations is needed to validate the effectiveness of these herbs.

The limitations of this study include the differences in diet and psychological and mental states of the participants at the time of answering questions, which was out of the researchers’ control. It is also important to consider that environmental and genetic factors may affect infant weight gain. The study used randomisation to adjust for this. One of the strengths of the study was the recommendation to use herbal medicines that caused no complications, increased breast milk supply in lactating women and reduced the incidence of diseases in infants. Being a triple-blind study was another strength of this study as this helped improve the accuracy of the findings.

## Conclusion

Green cumin is effective in improving the indicators of breast milk adequacy in lactating women, and it is more effective than nettle in this respect. Hence, it is suggested that the Ministry of Health authorities in the areas of maternal and infant health and midwifery recommend the use of green cumin drops as a galactogogue to mothers, midwives and specialists.

## Figures and Tables

**Table 1:** Characteristics of the parents and their infant across two experimental groups and a placebo group (N = 117).

Characteristic	n (%)			*P* value
	Experimental groups		Placebo group	
	Cumin (n = 39)	Nettle (n = 39)	(n = 39)	
**Mean age in years ± SD**	25.8 ± 7.06	27.3 ± 4.48	29.05 ± 6.73	0.293*
**Mean weight in kg ± SD**	77.15 ± 9.63	78.05 ± 9.38	81.38 ± 12.39	0.170*
**Mean height in cm ± SD**	166.2 ± 3.32	165.4 ± 3.37	163.06 ± 17.3	0.397*
**Mean infant weight in g ± SD**	3323.78 ± 337.4	3301.78 ± 377.05	3281.92 ± 302.41	0.606*
**Mean infant height in cm ± SD**	49.77 ± 1.33	50.06 ± 1.93	49.046 ± 1.75	0.224*
**Mean circumference of infant’s head in cm ± SD**	34.75 ± 1.24	34.87 ± 1.25	34.67 ± 1.19	0.617*
**Mother’s education level**				0.343†
Illiterate	1 (2.6)	4 (10.3)	2 (5.1)	
Elementary	2 (5.1)	0 (0.0)	3 (7.7)	
Guidance	6 (15.4)	4 (10.3)	13 (33.3)	
Diploma	26 (66.7)	20 (51.3)	13 (33.3)	
University	4 (10.3)	11 (28.2)	8 (20.5)	
**Father’s education level**				0.392†
Illiterate	1 (2.6)	3 (7.7)	1 (2.6)	
Elementary	5 (12.8)	0 (0.0)	5 (12.8)	
Guidance	10 (25.6)	6 (15.4)	10 (25.6)	
Diploma	16 (41.0)	19 (48.7)	16 (41.0)	
University	7 (17.9)	11 (28.2)	7 (17.9)	
**Economic situation**				0.237†
Weak	6 (15.4)	7 (17.9)	2 (5.1)	
Medium	28 (71.8)	25 (64.1)	28 (71.8)	
Strong	5 (12.8)	7 (17.9)	9 (23.1)	
**Number of deliveries**				0.382†
0	26 (66.7)	13 (33.3)	16 (41.0)	
1	5 (12.8)	21 (53.8)	12 (30.8)	
2	7 (17.9)	4 (10.3)	6 (15.4)	
>2	1 (2.6)	1 (2.6)	5 (12.8)	
**Voluntary delivery**				0.071†
Yes	32 (82.1)	35 (89.7)	25 (64.1)	
No	7 (17.9)	4 (10.3)	14 (35.9)	
**Type of delivery**				0.087†
NVD	11 (28.2)	13 (33.3)	26 (66.7)	
CS	28 (71.8)	26 (66.6)	13(33.4)	

SD = standard deviation; NVD = normal vaginal delivery; CS = Caesarean section.

* Using one way analysis of variance test;

† Using Chi-squared test.

**Table 2:** Comparison of feeding frequency, number of wet diapers, frequency of infant defecation and diaper weight in different groups before intervention.

Variable Group	Mean ± SD				
	Infant weight in g	Feeding frequency	Number of wet diapers	Frequency of defecation	Diaper weight in g
Placebo	3289.60 ± 64.30	7.12 ± 1.18	3.25 ± 1.03	0.47 ± 0.50	1091.28 ± 127.34
Cumin	3229.62 ± 75.30	7.80 ± 1.10	3.45 ± 1.03	0.50 ± 0.50	1103.52 ± 126.44
Nettle	3301.62 ± 45.31	7.84 ± 1.16	3.40 ± 1.18	0.52 ± 0.50	1107.10 ± 126.58
***P*** ** value** *	**0.891**	**0.921**	**0.783**	**0.898**	**0.841**

SD = standard deviation.

* Using one way analysis of variance test.

**Table 3:** Comparison of feeding frequency, number of wet diapers, frequency of defecation and diaper weight in different groups after intervention.

Variable Group	Mean ± SD				
	Infant weight in g	Feeding frequency	Number of wet diapers	Frequency of defecation	Diaper weight in g
Placebo	3519.97 ± 64.95	10.64 ± 0.96	6.80 ± 0.68	2.00 ± 0.78	1140.75 ± 151.62
Cumin	4031.55 ± 103.40	13.79 ± 0.86	13.67 ± 0.86	3.15 ± 0.66	1445.06 ± 148.99
Nettle	3863.67 ± 94.77	12.90 ± 0.68	10.70 ± 1.07	2.37 ± 0.58	1352.11 ± 157.50
***P*** ** value** *	**<0.001**	**<0.001**	**<0.001**	**<0.001**	**<0.001**

SD = standard deviation.

* Using one way analysis of variance test.
